# The effect of HNF4α knockout in beta cells is age and sex dependent

**DOI:** 10.1080/19382014.2025.2552549

**Published:** 2025-09-29

**Authors:** Catharina B. P. Villaca, Viviane R. Oliveira, Gustavo J. Santos, Fernanda Ortis

**Affiliations:** a Department of Cell and Developmental Biology, Institute of Biomedical Science (ICB), University of São Paulo (USP), São Paulo, Brazil; b Department of Physiological Sciences, Federal University of Santa Catarina (UFSC), Santa Catarina, Brazil; c National Institute on Science and Technology in Bioanalytic, INCTBio, Campinas, Brazil

**Keywords:** Diabetes *mellitus*, apoptosis, HNF4α, beta cell, dedifferentiation

## Abstract

HNF4α is important for beta cells' ability to adequately secrete insulin in response to glucose concentration and endoplasmic reticulum (ER) homeostasis. In humans, HNF4α mutations are responsible for Diabetes *mellitus* subtype MODY1, which has an age-determining onset. Additionally, in other forms of DM, there is evidence that sex can influence beta cell dysfunction, with possible involvement of ER stress pathways. Thus, we assessed the influence of sex and age on beta-cell dysfunction induced by HNF4α absence. We used an animal model with specific beta cells KO of HNF4α, induced after birth (Ins. CRE HNF4α^loxP/loxP^). Glucose intolerance is observed after 10 d of KO induction, at 50 d of age, with KO males (MKO) displaying more severe glucose intolerance than KO females (FKO). The percentage of insulin-positive cells in KO mice islets is lower compared to Control at all ages evaluated, with MKO mice showing a more pronounced decline at later ages compared to FKO. Both KO groups exhibited reduced beta cell mass and increased *α*-cell mass, which was more pronounced in MKO. ER stress was induced in both KO groups; however, ER stress-mediated apoptosis was observed only in MKO. FKO mice show evidence of beta cell differentiated state loss. In summary, beta cell loss in HNF4α-KO is influenced by sex and age, involves induction of ER stress, and is more severe in males, where ER stress-induced beta cell death is observed. Partial protection observed in females seems to involve dedifferentiation of beta cells.

## Graphical Abstract

**Figure:**
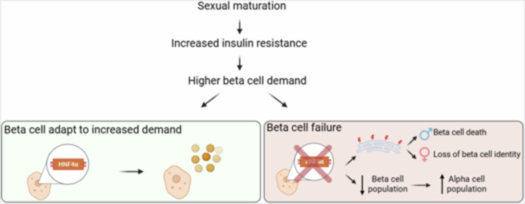

## Introduction

Diabetes *mellitus* (DM) is a set of metabolic disorders marked by chronic hyperglycemia resulting from impaired insulin action and/or secretion.^1^ These disorders include polygenic forms, such as type 1 and type 2 DM, as well as rarer monogenic forms,^1^ such as the Mature Onset Diabetes of the Young (MODY), classified into subtypes based on the specific gene mutation leading to DM.^2^^,^^3^ MODY1 is a subtype defined by HNF4α mutations,^3^^,^^4^ highlighting the critical role of this transcription factor for beta cell function and survival.^5–9^

The endoplasmic reticulum (ER) is crucial for the production of secreted proteins,^10^ such as insulin.^11^ During periods of elevated blood glucose, there is an increased demand for beta cells to produce and secrete insulin, which places a greater demand on the ER's protein-folding capacity.^12^ This heightened activity may lead accumulation of misfolded proteins within the ER lumen, a condition known as ER stress.^13^^,^^14^ In this context, the Unfolded Protein Response (UPR) is activated, restoring ER homeostasis in most cases.^14^^,^^15^ In beta cells, UPR activation allows adequate insulin secretion to match the demand for insulin secretion, and its subsequent deactivation is important for cellular stress recovery.^16^

Although the activation of the UPR in a cyclic manner is beneficial for beta cells' secretory capacity, its chronic activation, as observed during chronic hyperglycemia or insulitis, can switch on pro-apoptotic pathways.^14^^,^^17^^,^^18^ Persistent ER stress triggers beta cell apoptosis and impairs their adaptative response to increased metabolic demand, preventing the reestablishment of normoglycemia.^19–21^ ER stress-mediated apoptosis is an important component of DM development.^14^^,^^22^ There are evidences that HNF4α may modulate UPR pathways in beta cells.^23^^,^^24^ However, these findings disagree on whether HNF4α contributes to a pro-adaptative^23^ or a pro-apoptotic profile of UPR.^24^

This conflict can be in part due to the model used since Sato et al. used female mice of 45-week-old and Moore et al. used 11-week-old mice, with no information provided about the sex. It is known that beta cell function can be influenced by different mechanisms, such as the environment of the pancreatic islets^25^ as well as sex and age.^19^^,^^26^ For instance, estrogen has a protective effect on beta cell apoptosis mediated by ER stress, and older females lose this protection due to menopause, having similar outcomes as males.^27^

 In addition to apoptosis, it has been established that beta cell failure in DM may also occur by loss of differentiated status,^28^^,^^29^ as observed for glucotoxicity that affects oxidative stress, inflammation, and ER stress pathways^30^ or direct induction of ER stress,^31^ leading to the loss of beta cell mature phenotype.

Here, in a mouse model of HNF4α-specific beta cell knockout (KO), we investigate the influence of sex and age on beta cell UPR activation and dedifferentiation in the absence of HNF4α. Important to point out that this is not a model for MODY1, in which only one allele of HNF4α gene is mutated, but rather a model for evaluating the direct effect of HNF4α importance for beta cells.

## Materials and methods

### Ethical approval

The animal experiments conducted in this study were in accordance with local law and approved by the Animal Experimentation Ethics Committee (CEUA) of the Biomedical Sciences Institute (ICB) of the University of Sao Paulo, Brazil, under protocol number 9180111119.

### Animals

#### Origin, welfare, and breeding

InsCre HNF4α^loxP/loxP^ mice from “Biotério Setorial do Departamento de Biologia Estrutural e Funcional da Universidade Estadual de Campinas/SP” were adapted to and maintained in the animal housing facilities of the Department of Cell and Developmental Biology (ICB-USP). Mice had access to food (Nuvilab cr1, Nuvital Nutrientes LDTA, Curitiba) and water *ad libitum* and were maintained at a controlled temperature (22°C) in a 12−12 h light/dark cycle and maintained in collective cages (max 4/cage).

We utilized Ins1^CreERT2^^32^ to generate our inducible model of postnatal HNF4α knockout, with a C57BL/6 mice background. We utilized HNF4α^loxP/loxP^; InsCre^+/+^ and HNF4α^loxP/loxP^; and InsCre^−/−^ littermates as the experimental groups, knockout (KO) and control (Ctr), respectively. Although the absence of an HNF4α^wt/wt^; InsCre^+/+^​​​​ group represents a limitation, it does not compromise the validity of our findings, as the comparisons made are appropriate to address the objectives of this study. Mice were genotyped using primer sequences recommended by The Jackson Laboratories (Maine, USA).

#### Ages of interest

**Figure 1.**
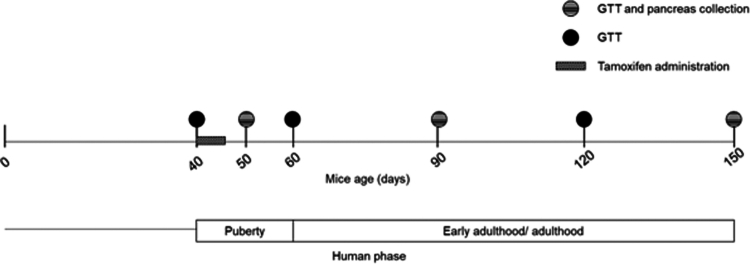
Experimental design. Scheme highlighting the key mice ages selected for the study and the experiments performed and their correlation with human ages. GTT: glucose tolerance test.

For the evaluation of islet cells, we selected 50 d (10 d after HNF4α-KO induction), 90, and 150 d of age (Figure 1), equivalent to early adulthood in humans. During this period, beta-cell dysfunction onset due to MODY1 is associated with hyperglycemia.^3^

 As age itself might affect glucose handling,^33^ we also evaluated glucose tolerance at 40 d (before induction of the KO), 60 d (final stages of puberty), and 120 d (midpoint) of age in addition to 50, 120, and 150 d of age (Figure 1). We weighed the mice weekly on the same day and at the time of the glucose tolerance test (GTT) assay.

We performed three independent experiments with five animals in each group (FCtr, FKO, MCtr, and MKO). Each experimental sample was collected at one of our time points of interest.

#### Tamoxifen administration for Knockout induction

Tamoxifen (#T5648, Sigma-Aldrich), diluted in sesame oil (#3547-0, Sigma-Aldrich), was administered by intraperitoneal injection to mice from both experimental groups (KO and Ctr) for 5 d (75 mg/kg) (Figure 1) as previously described.^23^

### Glucose tolerance test (GTT)

Animals were fasted overnight for 10–12 h, and fasting blood glucose (time 0) was evaluated through a blood sample obtained from the caudal vein measured in an Accu-Check glucometer (Roche). Then, glucose solution (1.25 g/kg) was administered intraperitoneally, and blood glucose was evaluated at 15, 30, 60, 90, and 120 min. This test was performed on animals at 40, 50, 60, 90, 120, and 150 d of age (Figure 1).

### Histological analysis

#### Pancreas processing and hematoxylin and eosin staining

At 50, 90, and 150 d of age, the pancreata were collected and processed for histological assays, sectioned, and stained with hematoxylin and eosin as previously described.^34^

#### Immunohistochemistry

The tissues were prepared for immunohistochemistry assays as previously described,^34^ and primary and secondary antibodies were diluted in TBS + BSA 3%. The stain was developed with diaminobenzidine (DAB) and hydrogen peroxide (Synth) against the stain with Mayer's hematoxylin and assembled in Damar gum. The primary antibodies used were anti-HNF4α (1:50, C11F12, Cell Signaling Technology), anti-insulin (1:100, sc-8, Santa Cruz) and anti-glucagon (1:200, L16G, Immunity Biotechnology), and the secondary antibody used was horseradish peroxidase-conjugated anti-rabbit IgG (1:200; MJ163713; Thermo Fisher Scientific; Waltham, MA, USA) or anti-mouse IgG (1:75; 31430; Santa Cruz Biotechnology).

#### Immunofluorescence

The tissue sections were processed as previously described.^34^ Mounting was done with Fluoroshield with DAPI (F6057; Sigma Aldrich). Slides were stored in the freezer for a maximum of 1 week before microscopic photography.

Slides were immunostained with anti-CHOP (1:100, Cell Signaling Technology), anti-XBP1-s (1:50, 40435S, Cell Signaling Technology), anti-GRP78 (1:20, PA1104A, Invitrogen), anti-Glut-2 (1:10, BS-10379R, Thermo Fisher), anti-PAX-4 (1:50, PA1108, Invitrogen), anti-NGN3 (1:100, BS-0922 R, Invitrogen) or anti-SOX9 (1:150, PA581966, Invitrogen) in combination with anti-insulin (1:100, sc-8, Santa Cruz or 1:100, 53-9-2, Thermo Fisher) and the corresponding secondary antibody anti-mouse (1:100, A1100 Invitrogen) or anti-rabbit (1:100, A11036 Invitrogen).

#### Islet image acquisition and quantification

All slides were documented using photomicrographs with Zeiss Axioskop 2. HE staining was documented with a 5× magnification objective to determine the section area, and 40× to determine the islet area, circularity, and the relative number of nuclei per islet. The immunostains were photographed at 40X magnification.

The slides and the images were independently evaluated by two individuals, one aware and one unaware of sample identity. The agreement between the findings obtained by the two observers was at least 90%. Quantification was performed using the “QuPath” software.^35^

The islets were manually traced, creating regions of interest (ROIs). Images of islets not used for this study were used to determine a positive threshold value based on stain intensity for each of the stains of interest to determine positive labeling. For HNF4α, the threshold value was 0.75, insulin was 0.35, and glucagon was 0.55. Within the islet ROI, we utilized the predetermined intensity value to determine individual islet positivity to the target of interest. Additionally, for the insulin labeling, we measured the variation of insulin intensity labeling, as this measurement serves as a proxy for protein abundance, as previously described.^36^^,^^37^ This was calculated using the formula: H-Score = 3∗intense score + 2∗medium score + 1∗weak score, as previously established.^38^

From the quantification of alpha and beta cell masses, we calculated using the formula: mass = (total positive area of the marker)/(total area of the cut) × wet weight of the pancreas (modified from).^39^

The QuPath software (“object classification”) quantified the colocalization with insulin immunofluorescence assays. This function allows the evaluation of cell by the cell of positivity for insulin labeling and whether this is in combination with positivity for other proteins (CHOP, GRP78/BiP, XBP1-s, GLUT-2, PAX-4, NGN3, and SOX9). The data are expressed as the percentage of positive cells for the label of interest in relation to the total of positive cells for insulin.

#### Statistical analysis

Statistical analysis was performed using the GraphPad Prism v9 software. The results were analyzed by two-way ANOVA with multiple comparisons (Tukey post hoc test). Except for the area under the curve (AUC) of the GTTs analysis, a three-way ANOVA with multiple comparisons was used.

## Results

### Animal model characterization

**Figure 2.**
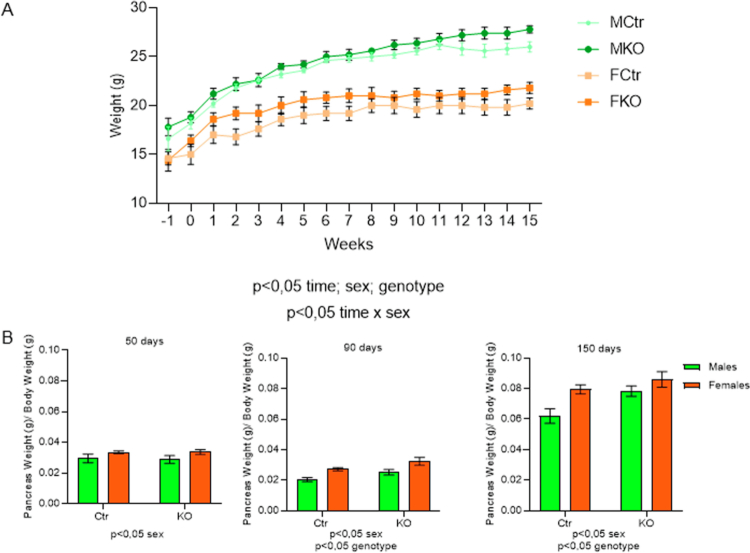
Body and pancreas weights. Weight gain during experimentation phase (week 0 being the tamoxifen administration week) (A) and pancreas wet weight after dissection (B) at 50, 90, and 150 d of age. The source of variation is reported under the graph (*n* = 5/group), and the data are shown as the mean ± SEM.

The percentage of islet cells positive for HNF4α immunostaining was reduced, around 80%, in KO mice compared to Ctr, 5 d after the last tamoxifen administration, as previously described.^8^ It is important to note that there was no difference between the sexes, with equal efficiency of KO in both females and males (Supplementary Figure 1). The HNF4α-KO body weight was not affected during the analyzed period. As expected, males had significantly higher body weight gain (Figure 2A).^40^ Females, independent of the genotype, had significantly higher proportional pancreas weights at all ages when compared to males (Figure 2B). Comparison between genotypes showed that KO had a higher proportional pancreas weight after 90 d compared to Ctr (Figure 2B).

**Figure 3.**
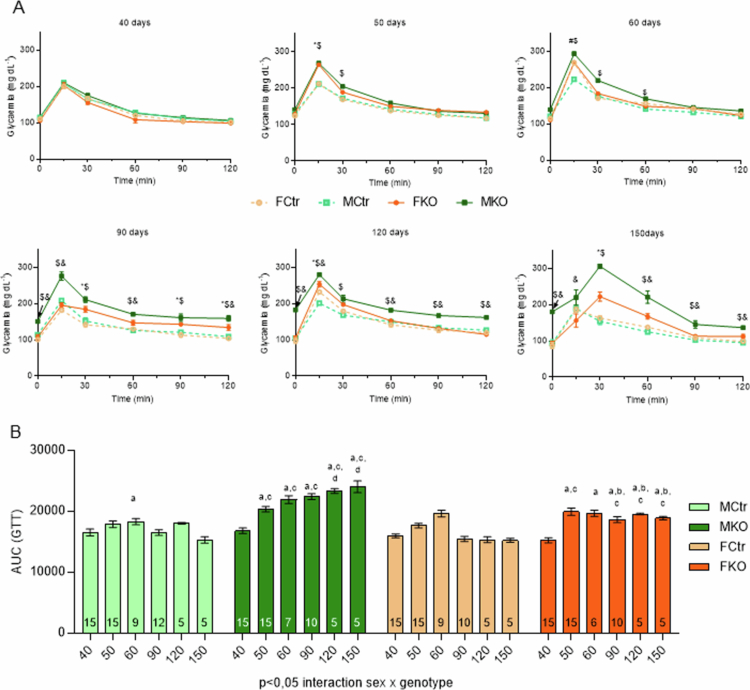
Glucose tolerance test. Glucose tolerance test at 40, 50, 60, 90, 120, and 150 d of age from all groups: FCtr (female control); MCtr (male control); FKO (female KO) and MKO (male KO). *p* < 0.05: * FKO vs FCtr, $ MKO vs MCtr, & MKO vs FKO, #MCtr vs FCtr (A). Area under the curve of these tests *p* < 0.05: a vs 40 d old (same group), b *p* < 0.05 vs male (same age and genotype), c vs Ctr (same age and sex), d *p* < 0.05 vs 50 d old (same group) (B). The source of variation is reported under the graph. *N* of each test is reported within the bars in B, and the data are shown as the mean ± SEM.

At 40 d of age, before KO induction, all groups had similar GTT curves (Figure 3A). Ten days after KO induction (50 d of age), KO animals of both sexes presented increased glycemia 15 min after glucose administration compared to the respective Ctr. At 30 min, only KO males MKO maintained this difference (Figure 3A). At a later age in puberty (60 d), only MKO presented higher glycemia at the first three points of the curve. Although, at this age, Ctr females (FCtr) presented higher blood glucose at 15 min, when compared to males of the same genotype, there is no difference between the KO groups (Figure 3A).

At the beginning of the young adult phase (90 d of age), MKO showed hyperglycemia at all points of the GTT, including during fasting, compared to the MCtr. In contrast, the KO females (FKO) only showed this increase at later points (after 90 min) (Figure 3A). This glucose intolerance observed at all time points evaluated in MKO mice persisted at 120 d of age and was still in the young adult phase; in contrast, FKO mice at 120 d of age presented higher glycemia only at 15 min (Figure 3A). In the final part of the young adult phase (at 150 d of age), there is a change in glucose handling for KO animals since the GTT curve peak that typically occurs at 15 min is observed at 30 min in this group, independent of sex. The curve remains the same in Ctr animals, demonstrating that the temporal change occurs in response to the absence of HNF4α (Figure 3A).

Next, we evaluated the temporal progression of sex impact in animals of different genotypes using the 3-factor analysis of the AUC to assess the effect of sex on glucose tolerance in function of knockout and time. Taken together, the changes in glucose handling demonstrated that MKO has reduced glucose tolerance compared to FKO after 60 d of age (Figure 3B). In addition, the MKO shows a progressive loss in glucose tolerance with age, which is not observed in the FKO (Figure 3B).

### Islet structure and cell populations

**Figure 4.**
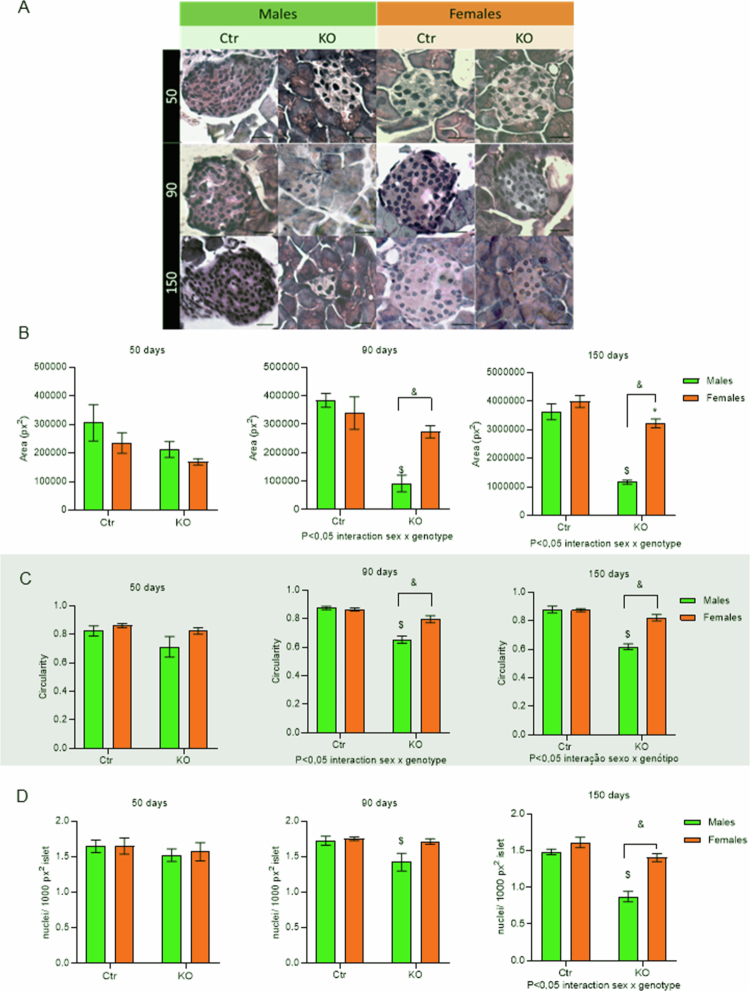
Islet morphometric parameters. Representative images of all 12 groups: four genotypes, FCtr (female control); MCtr (male control); FKO (female KO) and MKO (male KO), at 3 different ages (A). Quantification of average islet's area (B), circularity (C), and nuclei per islet (D). *N* = 5/group, data are shown as the mean ± SEM, *p* < 0.05: *FKO vs FCtr, $ MKO vs MCtr, and & FKO vs MKO, source of variation is reported under the graph. Scale bar: 50 μm.

Qualitative analysis of islet histology from the KO and control animals showed decrease in islet area of the KO animals at later ages (Figure 4A). Quantification of these parameters showed that a decrease in area (Figure 4B) and circularity (Figure 4C) in MKO animals was absent at 50 d and was observed only at later ages, 90 and 150-day-old animals, compared to both MCtr and FKO groups. For FKO, however, only at 150 d of age, there was a reduction of islet area compared to FCtr (Figure 4B), with no changes in islet circularity (Figure 4C). In agreement with these results, a reduction of islet cell number, evaluated by the number of nuclei/islets (Figure 4D), was observed in MKO islets when compared to MCtr at 90 and 150 d of age and only at 150 d when compared to FKO islets (Figure 4D). Thus, the influence of sex (*p* < 0.05 sex × interaction genotype) in the effect of HNF4α-KO in the islet area and circularity is already observed at 90 d of age (Figure 4).

**Figure 5.**
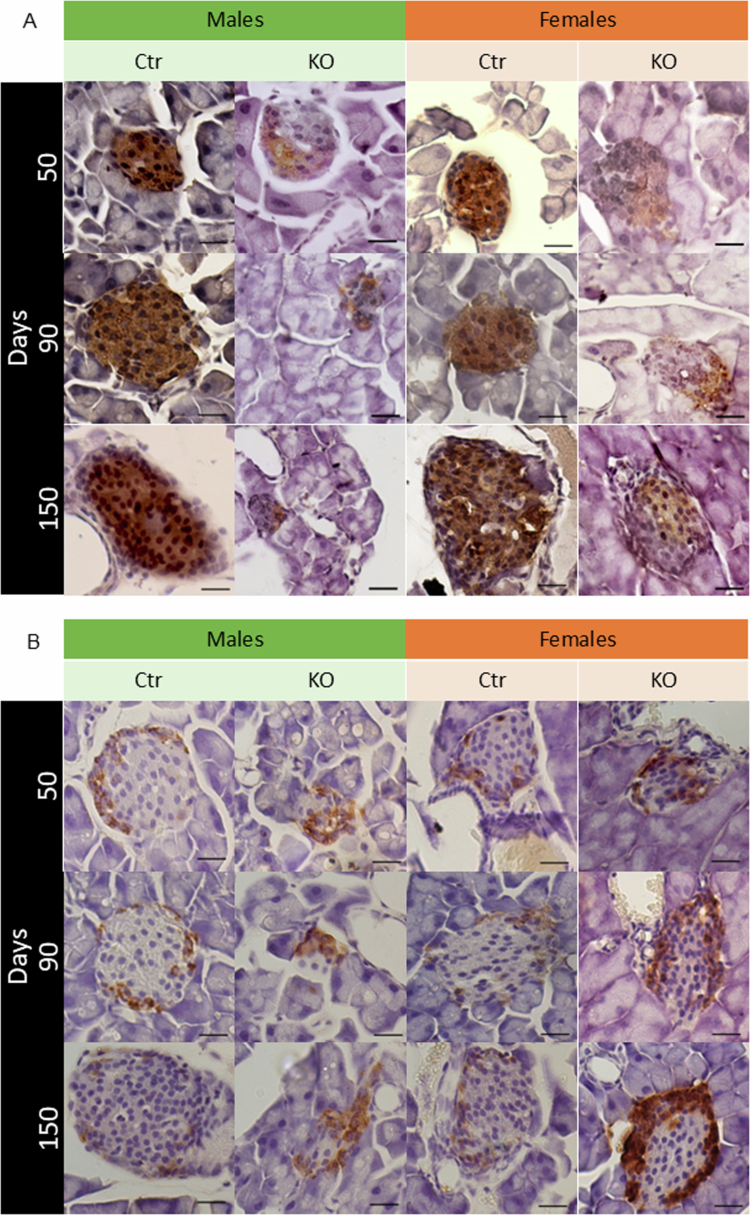
Insulin and glucagon stains. Representative images of all 12 groups: four genotypes, FCtr (female control); MCtr (male control); FKO (female KO) and MKO (male KO) at three different ages, showing the localization of the immunostaining and intensity of insulin (A) and glucagon (B). Scale bar: 50 μm.

**Figure 6.**
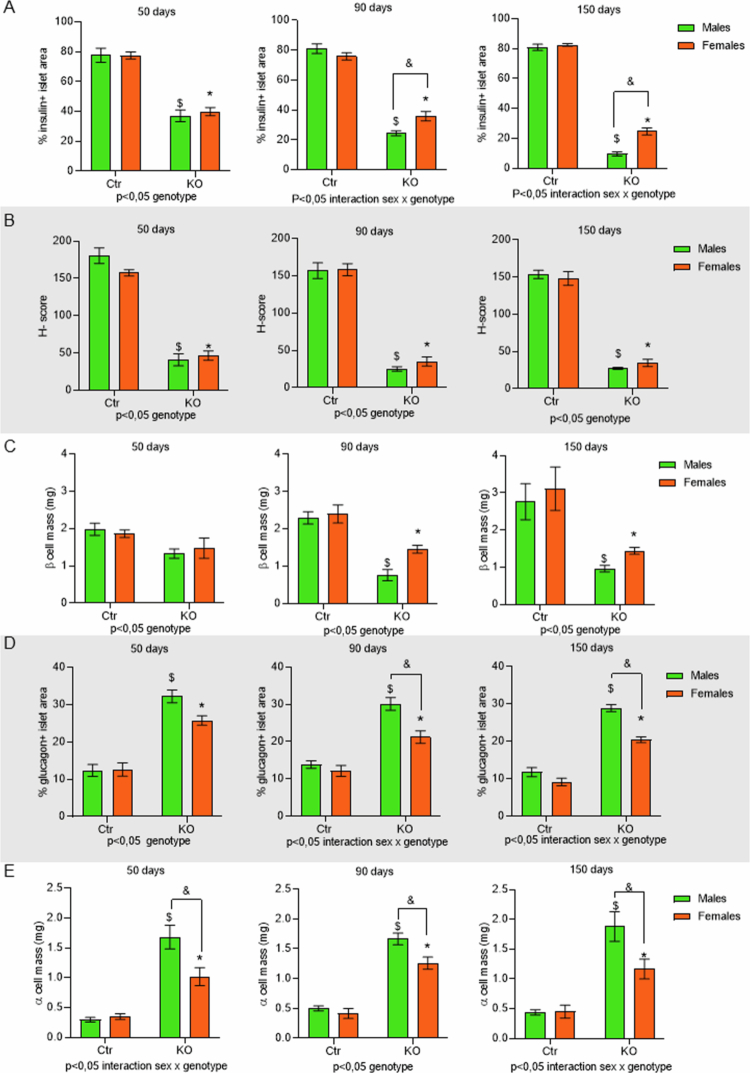
Quantification of insulin and glucagon immunostaining. Quantification of all 12 groups: four genotypes, FCtr (female control); MCtr (male control); FKO (female KO) and MKO (male KO), at three different ages for insulin area (A), H-score (B), beta cell mass (C), glucagon area (D), and alpha cell mass (E). *N* = 5 group, data are shown as the mean ± SEM, *p* < 0.05: *FKO vs FCtr, $ MKO vs MCtr, and & FKO vs MKO, source of variation is reported under the graph.

In both female and male control mice at all ages evaluated, insulin immunostaining is located in the center of the islet (Figure 5A) and glucagon in the periphery (Figure 5B), according to the expected cell distribution in rodents.^41^ In KO animals at 50, 90 and 150 d of age, insulin immunostaining is less evident, as shown by a decrease in the percentage of insulin-positive islet area, already at 50 d of age, compared to Ctr (Figure 6A). This loss of insulin-positive area in islets from KO animals is progressive with age and is more evident at 90 and 150 d. This progressive reduction is more intense in MKO, going from 37% insulin-positive islet cells at 50 d to 9% at 150 d of age, compared to FKO, going from 39% insulin-positive islet cells at 50 d to 24% at 150 d of age (Figures 5 and 6A). The intensity of insulin labeling (H-Score) is also significantly reduced in KO animals compared to Ctr animals at all ages, regardless of sex (Figure 6B). Finally, beta cell mass is reduced in KO animals compared to the respective Ctr from 90 d of age, regardless of sex (Figure 6C).

Glucagon immunostaining is more prominent in the KO than in the Ctr group (Figure 5B). The percentage of glucagon-positive cells per islet area was increased in KO groups compared to control animals at 50 d of age. From 90 d of age, this parameter starts to be influenced by sex, with MKO mice showing a significant increase in glucagon-positive cells compared to females of the same genotype (Figure 6D). Finally, the alpha cell mass is higher in the KO groups compared to the respective Ctrs at all ages, with MKO having a significant increase compared to FKO (Figure 6E).

### Endoplasmic reticulum stress markers in insulin-positive cells

**Figure 7.**
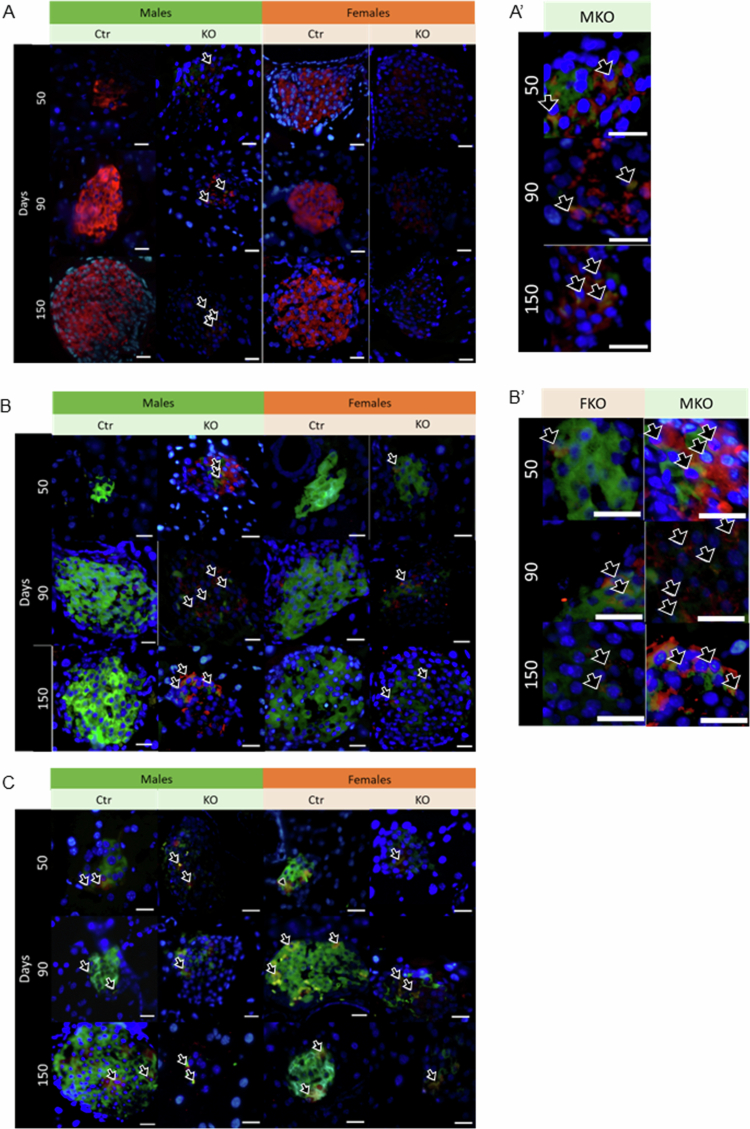
Endoplasmic reticulum stress markers colocalized with insulin. Representative images of all 12 groups: four genotypes FCtr (female control); MCtr (male control); FKO (female KO) and MKO (male KO) at three different ages, immunofluorescence showing the colocalization (yellow) of insulin (red) with CHOP (green) (A); or insulin (green) with GRP78 (red) (B); or XBP1-s (red) (C). On the right side of the panels there is a zoomed representative image of the colocalization observed in A (A′) and B (B′). Nuclei were stained with DAPI (blue). Scale bar: 50 μm.

**Figure 8.**
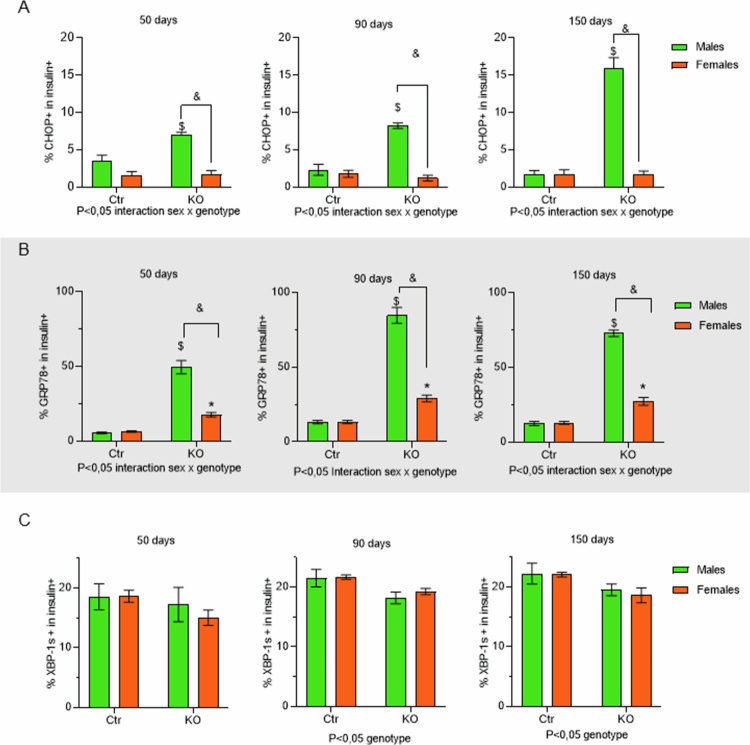
Quantification of the endoplasmic reticulum stress markers colocalized with insulin. Quantification of all 12 groups: four genotypes, FCtr (Female Control); MCtr (Male Control); FKO (Female KO) and MKO (Male KO) at three different ages, colocalization, expressed as positive percentage of either CHOP (A), GRP78 (B) or XBP1-s (C) positive cells in insulin + cells. *N* = 5 group, data are shown as the mean ± SEM, *p* < 0.05: *FKO vs FCtr, $ MKO vs MCtr, and & FKO vs MKO, source of variation is reported under the graph.

The colocalization of CHOP (green) with insulin (red) was exacerbated in islet cells from MKO animals at all ages analyzed, as highlighted in the right panel (Figure 7A). The quantification of this colocalization confirmed that only in MKO animals, the absence of HNF4α leads to an increase in CHOP in beta cells (Figure 8A).

HNF4α-KO also induced an increase in GRP78 expression on beta cells, observed by increased colocalization of GRP78 (red) and insulin (green) independent of sex (Figure 7B). However, at 50 d, the percentage of this colocalization was already higher in MKO than in FKO (Figure 8B). The increased expression of GRP78 in beta cells was progressive with age, being higher at 90 and 150 d in animals and more significant for MKO than for FKO (Figures 7B and 8B). Thus, sex and age influenced HNF4α-KO-induced GRP78 expression in beta cells.

Finally, XBP-1s (red) colocalization with insulin (green) was similar in all the groups evaluated (Figures 7 and 8C) at 50 d. However, in the KO groups, there was a reduction in this colocalization compared to Ctr group after 90 d, independent of sex (Figure 8C).

### Insulin-positive cells differentiated phenotype

**Figure 9.**
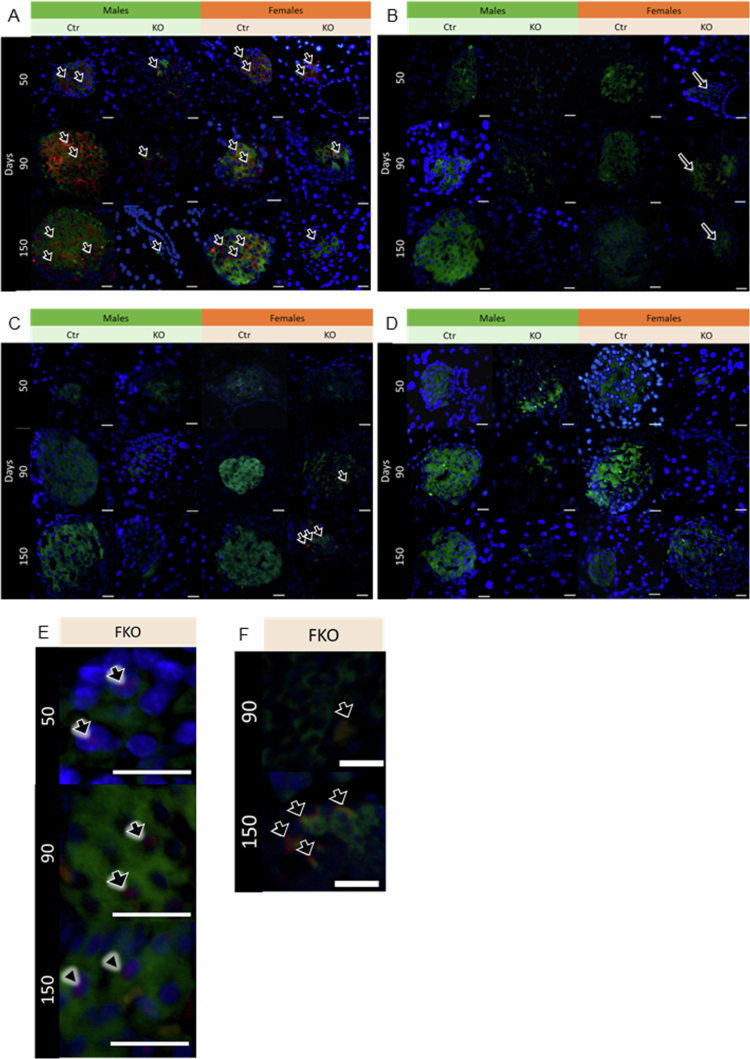
Beta cell phenotype markers colocalized with insulin. Representative images of all 12 groups: four genotypes FCtr, (female control); MCtr (male control); FKO (female KO) and MKO (male KO) at three different ages, immunofluorescence showing the colocalization of insulin (green) with either Glut-2 (red) (A), Pax-4 (red) (B), with highlights of nuclear colocalization (purple) on the bottom right panel (E). NGN3 (red) (C), with highlighted colocalization on the bottom right panel (F), or SOX9 (red) (D) with insulin (green). Nuclei were stained with DAPI (blue). Scale bar: 50 μm.

**Figure 10.**
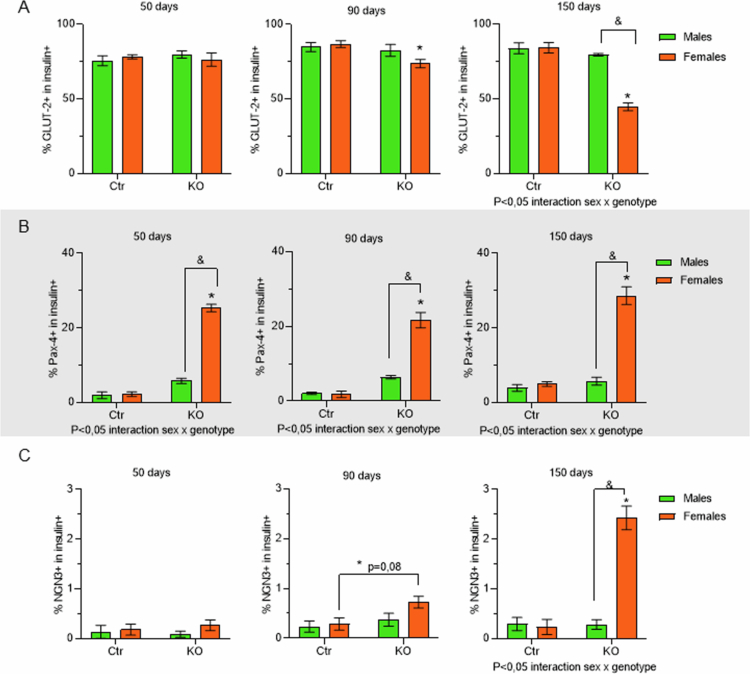
Quantification of the dedifferentiation markers colocalized with insulin. Quantification of all 12 groups: four genotypes, FCtr (Female Control); MCtr (Male Control); FKO (Female KO) and MKO (Male KO) at three different ages, colocalization, expressed as positive percentage of either Glut-2 (A), Pax-4 (B) or NGN3 (C) in insulin +  cells. *N* = 5 group, data are shown as mean ± SEM, p<0.05: *FKO vs FCtr, $ MKO vs MCtr, and & FKO vs MKO, source of variation is reported under the graph, source of variation is reported under the graph.

We next evaluated key differentiation markers that allow distinction of mature beta cells from different stages of endocrine progenitors. Glut-2 is expressed in mature beta cells, while PAX-4, NGN3 and SOX9 are expressed in different beta cells progenitors, from more mature to less mature stage, respectively.^42^ Glut-2 (red) is colocalized with insulin (green) in all groups and ages analyzed (Figure 9A). However, after 90 d of age, FKO showed lower expression of Glut-2. This reduction of Glut-2 expression in insulin-positive cells after 90 d of age was confirmed by quantification, and statistical analysis showed the influence of sex at 150 d of age (Figure 10A).

Expressive Pax-4 (red) nuclear colocalization (purple) in insulin (green)-positive cells was observed only in FKO animals at all ages analyzed (Figure 9B, highlighted in 9E). Thus, this increased nuclear presence of Pax-4 in beta cells induced by HNF4α absence is influenced by sex (Figure 10B).

Similarly, increased NGN3 (red) presence, which has a cytoplasmic localization, in insulin (green)-positive cells was observed only in FKO animals at 90 and 150 d of age (Figure 9C, highlighted in 9F). However, this increase was significantly higher only in FKO compared to FCtr group at 150 d of age. At that point, there was an influence of the sex, with MKO showing no alteration in NGN3 expression (Figures 9C and 10C). We observed no expression of SOX9 (red) in islet cells, immunostained with insulin (green), was similar for both sexes and genotypes (Figure 9D).

## Discussion

The absence of HNF4α leads to impaired insulin secretion and loss of functional beta cell mass.^5–9^^,^^43^ MODY1 is a monogenic form of DM due to mutations in the transcription factor HNF4α with onset after puberty.^3^^,^^4^ Here, we have demonstrated for the first time that beta cell outcome after HNF4α absence is influenced not only by age but also by sex. Thus, although male and female mice with beta cell-specific HNF4α knockout (MKO and FKO, respectively) become hyperglycemic, only MKO mice have worsening glucose intolerance dependent on age. We propose that the difference between sexes in glucose handling loss can be attributed to different outcomes of UPR activation in the beta cells of our KO mice. Our data suggest that the MKO have a chronic activation of UPR, leading to apoptosis. On the other hand, for FKO it does not progress to apoptosis, and instead induces loss of beta cell identity, an outcome of UPR activation previously described.^44^ In both cases, beta cell loss is exacerbated by age.

The rapid impairment of glucose tolerance due to HNF4α absence, observed here is in line with its essential role in beta cell insulin production and secretion.^6^^,^^8^ Interestingly, elevated fasting blood glucose—a marker of chronic hyperglycemia progression^45^—is observed only in MKO mice, suggesting that the beta cell loss observed in males has a more significant functional impact.

Although previous work evaluating the role of HNF4α in beta cell function observed loss of glucose tolerance without changes in the insulin-positive cell percentage,^8^ this study did not evaluate different ages and sex, which may explain the differences in our results. Further analysis of our KO mice islets revealed significant morphometric changes, with more expressive changes in circularity observed in MKO, presenting correlation with the more significant glucose tolerance dysfunction in this group, as it is observed that islet morphological changes may reflect a beta cell function change.^46^ Indeed, the organization of cell types in the islet and its cytoarchitecture play an essential role in beta cell function.^25^^,^^47^

Alterations in islet morphology suggest that overall islet cell interactions may be disturbed since the different islet cell types influence each other.^25^^,^^48^^,^^49^ Thus, we evaluated alpha cells distribution in the islet, as it is known that beta cells can influence alpha cell proliferation and function.^49^^,^^50^ Indeed, we observed impaired beta cell mass and insulin labeling with an increased proportion of glucagon-positive cells per islet, which could further contribute to overall hyperglycemia.

To elucidate the mechanism through which HNF4α absence could affect beta cell function, as previously observed,^8^ we evaluated the expression of UPR components. We have shown that the colocalization of GRP78, a key ER chaperone involved in UPR response,^14^ and insulin is increased in KO mice, with a notably higher, age dependent-increasing colocalization in MKO, correlating with glucose intolerance. Increased GRP78 expression is observed during ER stress in beta cells.^14^^,^^51^^,^^52^ This suggested that the absence of HNF4α leads to beta cell loss through altered UPR signaling. It has been shown that UPR signaling in beta cells has three outcomes: resolution of ER stress, apoptosis and loss of identity.^17^^,^^44^ As such, we investigated these outcomes in our KO mice. Interestingly, only MKO has increased CHOP colocalization with insulin-positive cells, indicating activation of the apoptotic pathway in beta cells.^21^^,^^53^ Thus, it may be possible that progressive beta cell loss in MKO occurs as a result of impaired beta cell ER function, due to HNF4α absence that together with progressive consequent hyperglycemia further aggravate the ER stress and beta cell loss via induction of ER apoptotic pathways.^17^^,^^18^^,^^22^

Analysis of UPR pathway components demonstrates a sex-based difference in UPR activation outcome. Although there was reduced beta cell sXBP1, a marker of adaptative UPR, in both sexes, only in males, was this accompanied by an increase in apoptotic marker, CHOP, and greater induction of GRP78. This suggests that in females the UPR adaptive response seems to be impaired, which may lead to loss of proper beta cell function^54^^,^^55^ and loss of beta cell identity.^31^^,^^44^ Indeed, proper sXBP1 expression is essential for maintaining beta cell identity^56^ and other components of the adaptative UPR.^44^^,^^57^^,^^58^

One key marker of beta cell mature state is Glut-2 expression,^59^^,^^60^ which is reduced in FKO mice. In addition, FKO beta cell shows an increase in PAX-4 expression at all ages analyzed. Acute expression of this transcription factor is associated with a greater ability to reestablish ER homeostasis,^61^ which may explain the lower activation of GRP78 and CHOP expression in FKO beta cells. However, continuous expression of PAX-4 can lead to the loss of insulin secretion, as it determines a less mature beta cell phenotype.^62^^,^^63^ This is further highlighted by the presence of NGN3-positive cells in FKO mice, which are first observed at 90 d and more clearly at 150 d of age, as the reappearance of NGN3-positive cells in postembryonic islets implies the loss of fully mature beta cell state.^64^^,^^65^ The absence of SOX9 expression may indicate the presence of cells with a phenotype between mature beta cells and endocrine progenitors.^66^^,^^67^ Interestingly, it has been described that beta cells that have this partial loss of identity can produce and secrete low volumes of insulin independently of changes in plasma glucose.^59^^,^^68^ Therefore, a partial loss of identity in the beta cells of FKO mice could explain their better glucose tolerance than MKO mice, which needs further studies.

There is evidence in the literature that DM2 mouse models, with the same background of our model (C57BL/6J), exhibit sex differences in insulin resistance, with females generally being more protected.^69^^,^^70^ This represents an interesting avenue for future investigations. However, in our study, we observed no significant differences between male and female mice in the control groups, only between HNF4α-KO mice. These findings highlight the need to consider sex as a biological variable and suggest that Hnf4α deletion may interact with intrinsic physiological differences in β-cell vulnerability. These differences between sexes are not related to the use of tamoxifen to induce HNF4α-KO, since we first confirmed that the KO induction efficiency was similar to that previously described in the literature^8^ for both sexes.

In conclusion, the absence of HNF4α leads to impaired glucose tolerance and loss of insulin-producing beta cells, influenced by sex and age. Beta cell loss in the KO groups may occur due to ER stress pathway alteration. Furthermore, it is possible to hypothesize that sex influences whether this dysfunction will be induced by the activation of proapoptotic pathways, as observed in males, or by beta cell dedifferentiation, as observed in females.

## Supplementary Material

Supplementary material Supplementary Figure 1: HNF4α immunohistochemistry. Representative images of all 12 groups (four genotypes at three different ages), arrows point to some of the positive nuclei (A), grayscale of DAB filter (B), and quantification of average positive nuclei per islet (C). *N* = 5 groups; the data is shown as mean + SEM. FCtr (female control); MCtr (male control); FKO (female KO) and MKO (male KO). *p* < 0.05: *FKO vs FCtr, $ MKO vs MCtr and FKO vs MKO, source of variation is reported under the graph scale bar: 50 μm.

Graphical abstract v2 Graphical abstract v2

graphical_abstract graphical_abstract

## References

[1] ADA. 2. Classification and diagnosis of diabetes. Diabetes Care. 2015;38(Suppl 1):S8–S16. doi: 10.2337/dc15-S005. 25537714

[2] Ahmet A, Gönül Ç, Ayhan A, Ece B. Maturity-onset diabetes of the young (MODY): an update. J Pediatr Endocrinol Metab. 2015;28(3–4):251–263. doi: 10.1515/jpem-2014-0384. 25581748

[3] Fajans SS. Maturity-onset diabetes of the young (MODY). Diabetes Metab Rev. 1989;5(7):579–606. doi: 10.1002/dmr.5610050705. 2689121

[4] Polonsky KS. The β-cell in diabetes: from molecular genetics to clinical research. Diabetes. 1995;44(6):717. doi: 10.2337/diab.44.6.705. 7789637

[5] Ellard S. Hepatocyte nuclear factor 1 alpha (HNF-1 alpha) mutations in maturity-onset diabetes of the young. Hum Mutat. 2000;16(5):377–385. doi: 10.1002/1098-1004(200011)16:5<377:AID-HUMU1>3.0.CO;2-2. 11058894

[6] Stoffel M, Duncan SA. The maturity-onset diabetes of the young (MODY1) transcription factor HNF4alpha regulates expression of genes required for glucose transport and metabolism. Proc Natl Acad Sci USA. 1997;94(24):13209–13214. doi: 10.1073/pnas.94.24.13209. 9371825 PMC24288

[7] Gupta RK, Gao N, Gorski RK, White P, Hardy OT, Rafiq K, Brestelli JE, Chen G, Stoeckert CJ, Jr, Kaestner KH. Expansion of adult beta-cell mass in response to increased metabolic demand is dependent on HNF-4alpha. Genes Dev. 2007;21(7):756–69. doi: 10.1101/gad.1535507. 17403778 PMC1838528

[8] Gupta RK, Vatamaniuk MZ, Lee CS, Flaschen RC, Fulmer JT, Matschinsky FM, Duncan SA, Kaestner KH. The MODY1 gene HNF-4alpha regulates selected genes involved in insulin secretion. J Clin Invest. 2005;115(4):1006–15. doi: 10.1172/JCI22365. 15761495 PMC1059446

[9] Barth R, Ruoso C, Ferreira SM, de Ramos FC, Lima FB, Boschero AC, Santos GJD. Hepatocyte nuclear factor 4-α (HNF4α) controls the insulin resistance-induced pancreatic β-cell mass expansion. Life Sci. 2022;289:120213–120213. doi: 10.1016/j.lfs.2021.120213. 34902439

[10] Wei J, Hendershot LM. Protein folding and assembly in the endoplasmic reticulum. EXS. 1996;77:41–55. doi: 10.1007/978-3-0348-9088-5_4. 8856968

[11] Dodson G, Steiner D. The role of assembly in insulin's biosynthesis. Curr Opin Struct Biol. 1998;8(2):189–94. doi: 10.1016/s0959-440x(98)80037-7. 9631292

[12] Liu M, Weiss MA, Arunagiri A, Yong J, Rege N, Sun J, Haataja L, Kaufman RJ, Arvan P. Biosynthesis, structure, and folding of the insulin precursor protein. Diabetes Obes Metab. 2018;20(Suppl 2):28–50. doi: 10.1111/dom.13378. 30230185 PMC6463291

[13] Arunagiri A, Haataja L, Cunningham CN, Shrestha N, Tsai B, Qi L, Liu M, Arvan P. Misfolded proinsulin in the endoplasmic reticulum during development of beta cell failure in diabetes. Ann N Y Acad Sci. 2018;1418(1):5–19. doi: 10.1111/nyas.13531. 29377149 PMC5934315

[14] Meyerovich K, Fernanda O, Florent A, Alessandra KC. Endoplasmic reticulum stress and the unfolded protein response in pancreatic islet inflammation. J Mol Endocrinol. 2016;57(1):R1–R17. doi: 10.1530/JME-15-0306. 27067637

[15] Lenghel A, Alina M, Andrei M, Ana-Maria V. What is the sweetest UPR flavor for the β-cell? That is the question. Front Endocrinol. 2020;11:614123. doi: 10.3389/fendo.2020.614123. PMC7891099 33613449

[16] Xin Y, Dominguez Gutierrez G, Okamoto H, Kim J, Lee AH, Adler C, Ni M, Yancopoulos GD, Murphy AJ, Gromada J. Pseudotime ordering of single human β-cells reveals states of insulin production and unfolded protein response. Diabetes. 2018;67(9):1783–1794. doi: 10.2337/db18-0365. 29950394

[17] Eizirik DL, Cnop M. ER stress in pancreatic beta cells: the thin red line between adaptation and failure. Sci Signal. 2010;3(110):pe7–pe7. doi: 10.1126/scisignal.3110pe7. 20179270

[18] Oslowski CM, Urano F. The binary switch between life and death of ER stressed beta cells. Curr Opin Endocrinol Diabetes Obes. 2010;17(2):107–112. doi: 10.1097/MED.0b013e3283372843. 20125004 PMC2898716

[19] Le May C, Chu K, Hu M, Ortega CS, Simpson ER, Korach KS, Tsai MJ, Mauvais-Jarvis F. Estrogens protect pancreatic beta-cells from apoptosis and prevent insulin-deficient diabetes mellitus in mice. Proc Natl Acad Sci U S A. 2006;103(24):9232–7. doi: 10.1073/pnas.0602956103. 16754860 PMC1482595

[20] Chan JY, Luzuriaga J, Maxwell EL, West PK, Bensellam M, Laybutt DR. The balance between adaptive and apoptotic unfolded protein responses regulates β-cell death under ER stress conditions through XBP1, CHOP and JNK. Mol Cell Endocrinol. 2015;413:189–201. doi: 10.1016/j.mce.2015.06.025. 26135354

[21] Hu H, Mingxing T, Chan D, Shengqing Y. The C/EBP homologous protein (CHOP) transcription factor functions in endoplasmic reticulum stress-induced apoptosis and microbial infection. Front Immunol. 2019;9:3083. doi: 10.3389/fimmu.2018.03083. 30662442 PMC6328441

[22] Eizirik DL, Cardozo AK, Cnop M. The role for endoplasmic reticulum stress in diabetes mellitus. Endocr Rev. 2008;29(1):42–61. doi: 10.1210/er.2007-0015. 18048764

[23] Moore BD, Jin RU, Lo H, Jung M, Wang H, Battle MA, Wollheim CB, Urano F, Mills JC. Transcriptional regulation of x-box-binding protein one (XBP1) by hepatocyte nuclear factor 4α (HNF4α) is vital to beta-cell function. J Biol Chem. 2016;291(12):6146–57. doi: 10.1074/jbc.M115.685750. 26792861 PMC4813565

[24] Sato Y, Hatta M, Karim MF, Sawa T, Wei FY, Sato S, Magnuson MA, Gonzalez FJ, Tomizawa K, Akaike T, et al. Anks4b, a novel target of HNF4α protein, interacts with GRP78 protein and regulates endoplasmic reticulum stress-induced apoptosis in pancreatic β-cells. J Biol Chem. 2012;287(27):23236–45. doi: 10.1074/jbc.M112.368779. 22589549 PMC3391104

[25] Brereton MF, Vergari E, Zhang Q, Clark A. Alpha-, Delta- and PP-cells: are they the architectural cornerstones of islet structure and co-ordination? J Histochem Cytochem. 2015;63(8):575–91. doi: 10.1369/0022155415583535. 26216135 PMC4530398

[26] Aguayo-Mazzucato C, van Haaren M, Mruk M, Lee TB, Jr, Crawford C, Hollister-Lock J, Sullivan BA, Johnson JW, Ebrahimi A, Dreyfuss JM, et al. β Cell aging markers have heterogeneous distribution and are induced by insulin resistance. Cell Metab. 2017;25(4):898–910.e5. doi: 10.1016/j.cmet.2017.03.015. 28380379 PMC5471618

[27] Basu A, Dube S, Basu R. Men are from mars, women are from venus: sex differences in insulin action and secretion. Adv Exp Med Biol. 2017;1043:53–64. doi: 10.1007/978-3-319-70178-3_4. 29224090

[28] Bensellam M, Jonas JC, Laybutt DR. Mechanisms of β-cell dedifferentiation in diabetes: recent findings and future research directions. J Endocrinol. 2018;236(2):R109–R143. doi: 10.1530/JOE-17-0516. 29203573

[29] Patel S, Remedi MS. Loss of β-cell identity and dedifferentiation, not an irreversible process? Front Endocrinol. 2024;15:1414447. doi: 10.3389/fendo.2024.1414447. PMC11194313 38915897

[30] Khin PP, Lee JH, Jun HS. A brief review of the mechanisms of β-cell dedifferentiation in type 2 diabetes. Nutrients. 2021;13(5), 10.3390/nu13051593. PMC8151793 34068827

[31] Zhang Y, Sui L, Du Q, Haataja L, Yin Y, Viola R, Xu S, Nielsson CU, Leibel RL, Barbetti F, et al. Permanent neonatal diabetes-causing insulin mutations have dominant negative effects on beta cell identity. Mol Metab. 2024;80:101879–101879. doi: 10.1016/j.molmet.2024.101879. 38237895 PMC10839447

[32] Thorens B, Tarussio D, Maestro MA, Rovira M, Heikkilä E, Ferrer J. Ins1(Cre) knock-in mice for beta cell-specific gene recombination. Diabetologia. 2015;58(3):558–65. doi: 10.1007/s00125-014-3468-5. 25500700 PMC4320308

[33] Hoffman RP, Vicini P, Sivitz WI, Cobelli C. Pubertal adolescent male-female differences in insulin sensitivity and glucose effectiveness determined by the one compartment minimal model. Pediatr Res. 2000;48(3):384–8. doi: 10.1203/00006450-200009000-00022. 10960508

[34] Villaça CD, Cavalcante de Paula C, Cruz de Oliveira N, Vilas-Boas EA, Carolina dos Santos-Silva J, Ferreira de Oliveira S, Abdulkader F, Ferreira SM, Ortis F. Beneficial effects of physical exercise for β-cell maintenance in a type 1 diabetes mellitus animal model. Exp Physiol. 2021;106(7):1482–1497. doi: 10.1113/EP088872. 33913203

[35] Bankhead P, Loughrey MB, Fernández JA, Dombrowski Y, McArt DG, Dunne PD, McQuaid S, Gray RT, Murray LJ, Coleman HG, et al. QuPath: open source software for digital pathology image analysis. Sci Rep. 2017;7(1):16878–16878. doi: 10.1038/s41598-017-17204-5. 29203879 PMC5715110

[36] Braun M, Kirsten R, Rupp NJ, Moch H, Fend F, Wernert N, Kristiansen G, Perner S. Quantification of protein expression in cells and cellular subcompartments on immunohistochemical sections using a computer supported image analysis system. Histol Histopathol. 2013;28(5):605–610. doi: 10.14670/HH-28.605. 23361561

[37] Meyerholz DK, Beck AP. Principles and approaches for reproducible scoring of tissue stains in research. Lab Invest. 2018;98(7):844–855. doi: 10.1038/s41374-018-0057-0. 29849125

[38] Bankhead P, Fernández JA, McArt DG, Boyle DP, Li G, Loughrey MB, Irwin GW, Harkin DP, James JA, McQuaid S, et al. Integrated tumor identification and automated scoring minimizes pathologist involvement and provides new insights to key biomarkers in breast cancer. Lab Invest. 2018;98(1):15–26. doi: 10.1038/labinvest.2017.131. 29251737

[39] Dos Santos C, Rafacho A, Ferreira SM, Vettorazzi JF, Dos Reis Araújo T, Mateus Gonçalves L, Ruhrmann S, Bacos K, Ling C, Boschero AC, et al. Excess of glucocorticoids during late gestation impairs the recovery of offspring's β-cell function after a postnatal injury. FASEB J. 2021;35(8):e21828–e21828. doi: 10.1096/fj.202100841R. 34325494

[40] Reed DR, Bachmanov AA, Tordoff MG. Forty mouse strain survey of body composition. Physiol Behav. 2007;91(5):593–600. doi: 10.1016/j.physbeh.2007.03.026. 17493645 PMC2085171

[41] Kim A, Kevin M, Junghyo J, German K, Pawel W, Manami H. Islet architecture: a comparative study. Islets. 2009;1(2):129–136. doi: 10.4161/isl.1.2.9480. 20606719 PMC2894473

[42] Pan FC, Wright C. Pancreas organogenesis: from bud to plexus to gland. Dev Dyn. 2011;240(3):530–65. doi: 10.1002/dvdy.22584. 21337462

[43] de Ramos FC, Barth R, Santos MR, Almeida MDS, Ferreira SM, Rafacho A, Boschero AC, Dos Santos GJ. Hepatocyte nuclear factor 4-α is necessary for high fat diet-induced pancreatic β-cell mass expansion and metabolic compensations. Front Endocrinol (Lausanne). 2024;15:1511813–1511813. doi: 10.3389/fendo.2024.1511813. 39741884 PMC11685084

[44] Chen CW, Guan BJ, Alzahrani MR, Gao Z, Gao L, Bracey S, Wu J, Mbow CA, Jobava R, Haataja L, et al. Adaptation to chronic ER stress enforces pancreatic β-cell plasticity. Nat Commun. 2022;13(1):4621–4621. doi: 10.1038/s41467-022-32425-7. 35941159 PMC9360004

[45] Wysham C, Shubrook J. Beta-cell failure in type 2 diabetes: mechanisms, markers, and clinical implications. Postgrad Med. 2020;132(8):676–686. doi: 10.1080/00325481.2020.1771047. 32543261

[46] Jacques-Silva MC, MCorrea-edina M, Cabrera O, Rodriguez-Diaz R, Makeeva N, Fachado A, Diez J, Berman DM, Kenyon NS, Ricordi C, et al. ATP-gated P2X3 receptors constitute a positive autocrine signal for insulin release in the human pancreatic beta cell. Proc Natl Acad Sci U S A. 2010;107(14):6465–70. doi: 10.1073/pnas.0908935107. 20308565 PMC2851966

[47] Almaça J, Caicedo A, Landsman L. Beta cell dysfunction in diabetes: the islet microenvironment as an unusual suspect. Diabetologia. 2020;63(10):2076–2085. doi: 10.1007/s00125-020-05186-5. 32894318 PMC7655222

[48] Briant LJB, Reinbothe TM, Spiliotis I, Miranda C, Rodriguez B, Rorsman P. δ-Cells and β-cells are electrically coupled and regulate α-cell activity via somatostatin: β-to-δ gap junction coupling. J Physiol. 2018;596(2):197–215. doi: 10.1113/JP274581. 28975620 PMC5767697

[49] Watts M, Ha J, Kimchi O, Sherman A. Paracrine regulation of glucagon secretion: the β/α/δ model. Am J Physiol Endocrinol Metab. 2016;310(8):E597–E611. doi: 10.1152/ajpendo.00415.2015. 26837808 PMC4835945

[50] Mizukami H, Takahashi K, Inaba W, Tsuboi K, Osonoi S, Yoshida T, Yagihashi S. Involvement of oxidative stress-induced DNA damage, endoplasmic reticulum stress, and autophagy deficits in the decline of β-cell mass in Japanese type 2 diabetic patients. Diabetes Care. 2014;37(7):1966–74. doi: 10.2337/dc13-2018. 24705612

[51] Cardozo AK, Ortis F, Storling J, Feng YM, Rasschaert J, Tonnesen M, Van Eylen F, Mandrup-Poulsen T, Herchuelz A, Eizirik DL. Cytokines downregulate the sarcoendoplasmic reticulum pump Ca2+ ATPase 2b and deplete endoplasmic reticulum Ca2+, leading to induction of endoplasmic reticulum stress in pancreatic beta-cells. Diabetes. 2005;54(2):452–61. doi: 10.2337/diabetes.54.2.452. 15677503

[52] Rutkowski D, Kaufman RJ. A trip to the ER: coping with stress. Trends Cell Biol. 2004;14(1):20–28. doi: 10.1016/j.tcb.2003.11.001. 14729177

[53] Matsuda T, Kido Y, Asahara S, Kaisho T, Tanaka T, Hashimoto N, Shigeyama Y, Takeda A, Inoue T, Shibutani Y. Ablation of C/EBPbeta alleviates ER stress and pancreatic beta cell failure through the GRP78 chaperone in mice. J Clin Invest. 2010;120(1):115–26. doi: 10.1172/JCI39721. 19955657 PMC2798684

[54] Chan JY, Luzuriaga J, Bensellam M, Biden TJ, Laybutt DR. Failure of the adaptive unfolded protein response in islets of obese mice is linked with abnormalities in β-cell gene expression and progression to diabetes. Diabetes. 2013;62(5):1557–68. doi: 10.2337/db12-0701. 23274897 PMC3636637

[55] Herbert TP, Laybutt DR. A reevaluation of the role of the unfolded protein response in islet dysfunction: maladaptation or a failure to adapt? Diabetes. 2016;65(6):1472–1480. doi: 10.2337/db15-1633. 27222391

[56] Lee K, Chan JY, Liang C, Ip CK, Shi YC, Herzog H, Hughes WE, Bensellam M, Delghingaro-Augusto V, Koina ME, et al. XBP1 maintains beta cell identity, represses beta-to-alpha cell transdifferentiation and protects against diabetic beta cell failure during metabolic stress in mice. Diabetologia. 2022;65(6):984–996. doi: 10.1007/s00125-022-05669-7. 35316840 PMC9076738

[57] Back S, Donalyn S, JaeSeok H, Benbo S, Mark R, Junying W, Robert DG, Subramaniam P, Randal JK. Translation attenuation through EIF2α phosphorylation prevents oxidative stress and maintains the differentiated state in beta cells. Cell Metab. 2009;10(1):13–26. doi: 10.1016/j.cmet.2009.06.002. 19583950 PMC2742645

[58] Moin ASM, Butler AE. Alterations in beta cell identity in type 1 and type 2 diabetes. Curr Diab Rep. 2019;19(9):83–83. doi: 10.1007/s11892-019-1194-6. 31401713 PMC6689286

[59] Neelankal John A, Morahan G, Jiang F-X. Incomplete re-expression of neuroendocrine progenitor/stem cell markers is a key feature of β-cell dedifferentiation. J Neuroendocrinol. 2017;29(1). doi: 10.1111/jne.12450. 27891681

[60] Thorens B. GLUT2, glucose sensing and glucose homeostasis. Diabetologia. 2015;58(2):221–32. doi: 10.1007/s00125-014-3451-1. 25421524

[61] Mellado-Gil JM, Carmen NJ, Alejandro M, Ana Isabel A, Esther F, Nadia C, Petra IL, Bru-Tari E, de Gracia Herrera-Gómez I, López-Noriega L, et al. PAX4 preserves endoplasmic reticulum integrity preventing beta cell degeneration in a mouse model of type 1 diabetes mellitus. Diabetologia. 2016;59:755–765. doi: 10.1007/s00125-016-3864-0. 26813254 PMC4779135

[62] Hu He KH, Lorenzo PI, Brun T, Jimenez Moreno CM, Aeberhard D, Vallejo Ortega J, Cornu M, Thorel F, Gjinovci A, Thorens B, et al. In vivo conditional Pax4 overexpression in mature islet β-cells prevents stress-induced hyperglycemia in mice. Diabetes. 2011;60(6):1705–15. doi: 10.2337/db10-1102. 21521872 PMC3114382

[63] Lorenzo PI, Juárez-Vicente F, Cobo-Vuilleumier N, García-Domínguez M, Gauthier BR. The diabetes-linked transcription factor PAX4: from gene to functional consequences. Genes (Basel). 2017;8(3), 10.3390/genes8030101. PMC5368705 28282933

[64] Talchai C, Xuan S, Lin HV, Sussel L, Accili D. Pancreatic β cell dedifferentiation as a mechanism of diabetic β cell failure. Cell. 2012;150(6):1223–34. doi: 10.1016/j.cell.2012.07.029. 22980982 PMC3445031

[65] Watada H. Neurogenin 3 is a key transcription factor for differentiation of the endocrine pancreas. Endocr J. 2004;51(3):255–64. doi: 10.1507/endocrj.51.255. 15256770

[66] Kawaguchi Y. Sox9 and programming of liver and pancreatic progenitors. J Clin Invest. 2013;123(5):1881–6. doi: 10.1172/JCI66022. 23635786 PMC3635727

[67] Seymour PA. Sox9: a master regulator of the pancreatic program. Rev Diabet Stud. 2014;11(1):51–83. doi: 10.1900/RDS.2014.11.51. 25148367 PMC4295800

[68] Baeyens L, Bonné S, German MS, Ravassard P, Heimberg H, Bouwens L. Ngn3 expression during postnatal in vitro beta cell neogenesis induced by the JAK/STAT pathway. Cell Death Differ. 2006;13(11):1892–9. doi: 10.1038/sj.cdd.4401883. 16514419

[69] Kim B, Park ES, Lee JS, Suh JG. Sex-specific differences in glucose metabolism and pancreatic function in streptozotocin-induced diabetic mice: The protective role of estrogen. Biochem Biophys Res Commun. 2025;775:152176–152176. doi: 10.1016/j.bbrc.2025.152176. 40499498

[70] Racine KC, Iglesias-Carres L, Herring JA, Wieland KL, Ellsworth PN, Tessem JS, Ferruzzi MG, Kay CD, Neilson AP. The high-fat diet and low-dose streptozotocin type-2 diabetes model induces hyperinsulinemia and insulin resistance in male but not female C57BL/6J mice. Nutr Res. 2024;131:135–146. doi: 10.1016/j.nutres.2024.09.008. 39389000

