# Supplementary figures and images for: The effect of HNF4α knockout in beta cells is age and sex dependent
Source: Islets. 2025 Sep 29;17(1):2552549. doi: 10.1080/19382014.2025.2552549 (PMC12482440; doi:10.1080/19382014.2025.2552549)

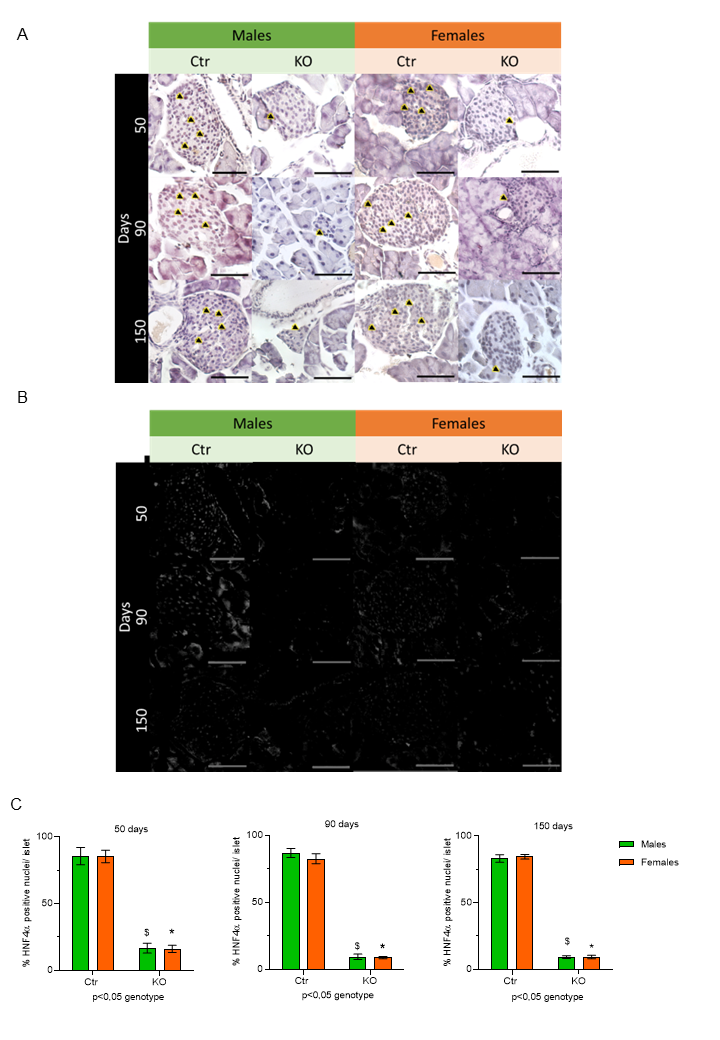

Supplement: Supplementary material — Supplementary Figure 1: HNF4α immunohistochemistry. Representative images of all 12 groups (four genotypes at three different ages), arrows point to some of the positive nuclei (A), grayscale of DAB filter (B), and quantification of average positive nuclei per islet (C). N = 5 groups; the data is shown as mean + SEM. FCtr (female control); MCtr (male control); FKO (female KO) and MKO (male KO). p < 0.05: *FKO vs FCtr, $ MKO vs MCtr and FKO vs MKO, source of variation is reported under the graph scale bar: 50 μm. [file KISL_A_2552549_SM4951.tif]

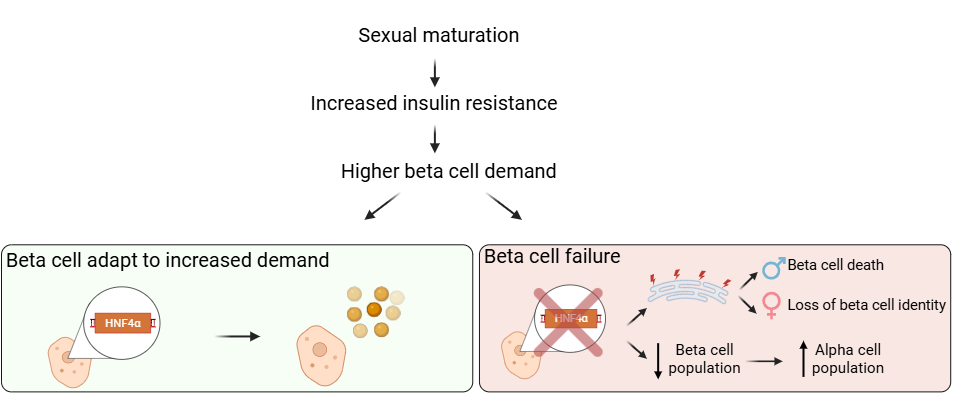

Supplement: Graphical abstract v2 [file KISL_A_2552549_SM4953.png]

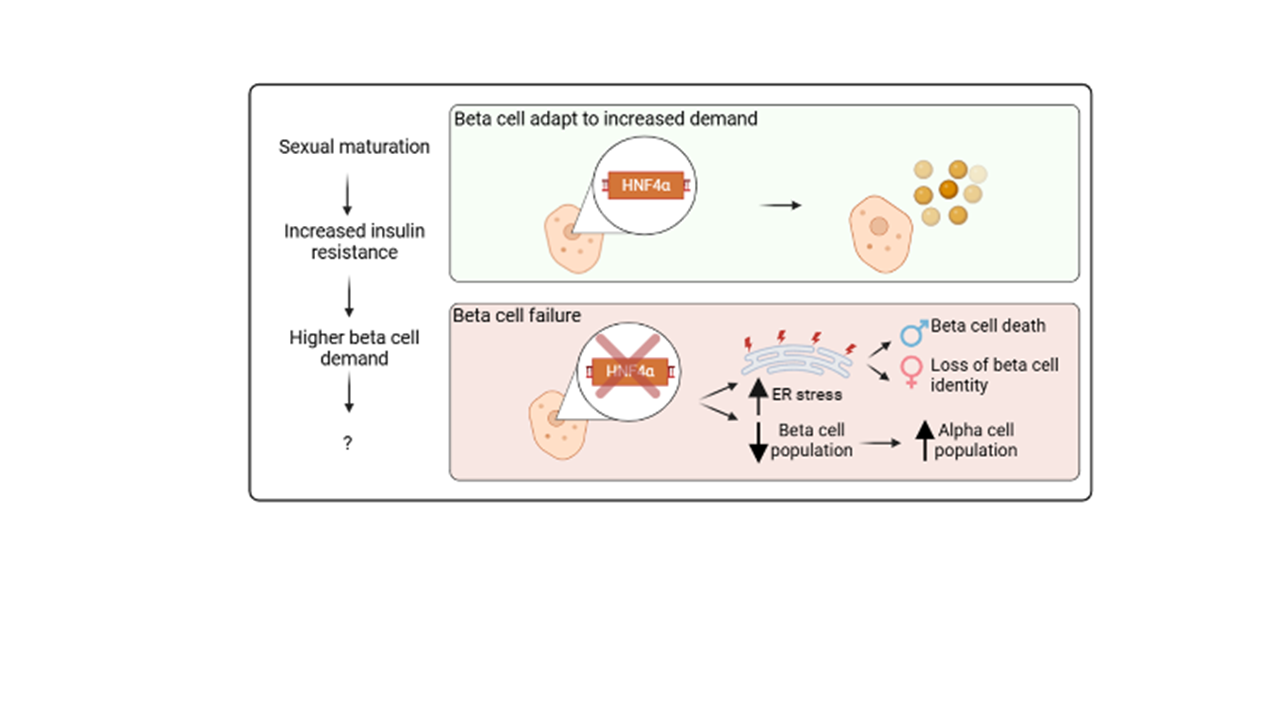

Supplement: graphical_abstract [file KISL_A_2552549_SM4952.tif]
